# Validation of the American version of the CareGiver Oncology Quality of Life (CarGOQoL) questionnaire

**DOI:** 10.1186/s12955-016-0487-6

**Published:** 2016-05-28

**Authors:** Sarah C. Kaveney, Karine Baumstarck, Patricia Minaya-Flores, Tarrah Shannon, Philip Symes, Anderson Loundou, Pascal Auquier

**Affiliations:** The Regional Cancer Center, 2500 West 12th Street, Erie, PA 16505 USA; EA3279, Self-perceived Health Assessment Research Unit, Aix Marseille Université, 27 bd Jean Moulin, Marseille, cedex 05 F-13385 France

**Keywords:** CarGOQoL, Caregivers, Cancer, Quality of life, Validation, Questionnaire

## Abstract

**Background:**

The CareGiver Oncology Quality of Life (CarGOQoL) questionnaire, a 29-item, multidimensional, self-administered questionnaire, was validated using a large French sample. We reported the linguistic validation process and the metric validity of the English version of CarGOQoL in the United- States.

**Methods:**

The translation process consisted of 3 consecutive steps: forward-backward translation, acceptability testing, and cognitive interviews. The psychometric testing was applied to caregivers of consecutive patients with representative cancers who were recruited from the Regional Cancer Center in northwestern Pennsylvania. All individuals completed the CarGOQoL at baseline, day- 30, and day- 90. Internal consistency, reliability, external validity, reproducibility, and sensitivity to change were tested.

**Results:**

The translated version was validated on a total of 87 American cancer caregivers. The dimensions of the CarGOQoL generally demonstrated a high internal consistency (Cronbach’s alpha > 0.70 for all but four domain scores). External validity testing revealed that the CarGOQoL index score correlated significantly with all SF-36 dimension scores except the physical composite score (Pearson’s correlation: 0.28–0.70). Reproducibility was satisfactory at day- 30 (intraclass correlation coefficient: 0.46–0.94) and day- 90 (0.43–0.92). Four specific dimensions of CarGOQoL showed responsiveness: the Psychological well-being, the Relationships with health care system, the Social support and the Finances.

**Conclusions:**

The American version of the CarGOQoL constitutes a useful instrument to measure QoL in caregivers of cancer patients in the United- States.

## Background

In recent decades, the progress in cancer treatment and cancer detection has been considerable. This has rendered cancer, in many cases, a chronic illness, thereby resulting in difficulties for patients and caregivers [1, 2]. It has been recognized that caregiving adversely effects the caregiver in terms of their health, emotional status [3–6], and quality of life (QoL) [7, 8].

Several groups have published detailed recommendations for the QoL assessment of caregivers. This is considered increasingly important with regard to evaluating the management of care provided to patients with chronic diseases [9–11]. QoL is commonly self-reported, and questionnaires must be robust, valid, and reliable, and must implement universally applied measures. Interviews with patients are commonly considered the best method to capture the patient’s perceptions.

Among the instruments used to measure the QoL of cancer caregivers [12], only three self-administered instruments were specifically developed for caregiver’ populations using standard methods: the Caregiver Quality of Life Index (CQLI) [13], the Caregiver Quality of Life Index-Cancer Scale (CQOLC) [14], and, most recently, the CareGiver Oncology Quality of Life (CarGOQoL) questionnaire [15]. The CQLI is a straightforward questionnaire that was validated using a sample of five subjects, who were asked only to indicate the relevance of predefined items. The CQOLC, which has been more thoroughly developed, was based on a mixed approach combining interviews with patient-caregiver dyads and experts’ points of view and was validated using a homogeneous sample comprised only of spouses. The CarGOQoL is the only questionnaire that meets the following criteria: i) based on the caregivers’ exclusive point of view (involving the content analysis of 77 face-to-face semi-structured interviews performed by experienced professionals), which is now recognized as the best approach to develop a questionnaire based on a clear conceptual basis for QoL [16–18]; ii) validated in a large heterogeneous sample of caregivers including partners, parents and children; and iii) capturing specific dimensions, such as self-esteem or private life. However, the CarGOQoL is solely available in the French language. We report the linguistic validation process and the metric validity of the English version of the CarGOQoL in the United-States.

## Methods

### Sample

Study participants were required to be: at least 18 years of age, designated by the patient as a ‘natural caregiver’ (‘the non-institutional relative/person who is taking care most of me and who is not paid to provide care’), able to speak/read English, and free from cancer comorbidity. Patients were selected from a community oncology practice in northwestern Pennsylvania (the Regional Cancer Center). Patients were diagnosed with either localized or metastatic primary cancer (leukemia, breast cancer, urologic cancer, melanoma, digestive cancer, lung cancer, and others). Caregivers were enrolled between July 2013 and February 2015.

### Ethics, consent and permissions

The participants all signed a written consent. The study was approved by The Saint Vincent Institutional Review Board (April 18, 2013; office number 814-452-5272).

### Study design and data collection

Caregivers were evaluated upon enrolment and were re-evaluated at 1 and 3 months. General characteristics related to the caregiver and the patient were collected upon inclusion. Self-administered survey materials were handed out and completed by the caregivers during the 3 evaluation times. These materials included the CarGOQoL questionnaire, the generic 36 Item Short Form (SF-36) questionnaire, and two visual analogic scales (VAS) to assess QoL and burden on a scale from 0 to 10. At the 1- and 3-month evaluations, caregivers were asked closed-ended questions about important (negative and/or positive) changes in their own lives since enrolment and about their perceptions of changes in the patient’s health (improved/stable/deteriorated).

- **General characteristics**

The following parameters were collected from the caregivers: gender, age, marital status, nature of the relationship with the patient, and caregiving duration. The following parameters were collected from the patients: gender, age, cancer localization, disease duration, stage, performance status.

- **Quality of life: CareGiver Oncology Quality of Life questionnaire and the short-form 36**

The CarGOQoL is a well-validated specific questionnaire for caregivers of cancer patients in France [15] that includes 29 questions describing 10 dimensions: psychological well-being, burden, relationship with health care, administration and finances, coping, physical wellbeing, self-esteem, leisure time, social support and private life. An index was computed. SF-36 questionnaire comprises 36 items that are used to calculate the following eight scale scores: physical functioning, social functioning, role–physical, role–emotional, mental health, vitality, bodily pain, and general health [19]. Two composite summary measures were also calculated: the Physical Component Summary (PCS) and the Mental Component Summary (MCS) scores. The PCS and MCS scores were norm-based using a linear T-score transformation with a mean of 50 and a standard deviation (SD) of 10. Both the CarGOQoL and the SF-36 questionnaires yielded scores on a 0–100 scale, where 0 represents the lowest QoL level and 100 represents the highest QoL level.

### General organization

The development and linguistic validation of a questionnaire should be based on a unique methodology and acknowledged by health authorities, ethics’ committees, and researchers in the field [20]. Two main steps were organized as follows: 1. the translation and the cultural adaptation process;and 2. psychometric testing. The two steps were planned under the coordination of a team that included the American clinical partners (two members of the Regional Cancer Center in northwestern Pennsylvania, United States of America) and the French developers of the CarGOQoL (two members of the Self-perceived Health Assessment Research Unit, Aix-Marseille University, Marseille, France).

- **Translation and cultural adaptation process**

The developers (PM, PA) provided a conceptual definition of the original items (termed “list of concepts”) to clarify the notions investigated in each item of the original French questionnaire. This list allowed for clarifying the notions investigated in each item of the original questionnaire in order to enhance harmonization across all language versions. The translation and the cultural adaptation processes were organized into several steps. First, forward translation of the CarGOQoL questionnaire from French into the target language (English) was performed by two native English speakers who were also fluent in French. Any differences between the 2 translated versions were discussed by the translators and the developers, thereby ensuring conceptual equivalence. A reconciled, harmonized, and agreed upon forward-translated version of the CarGOQoL was produced. Second, backward translation of this version into English was performed by two native French speakers who were also fluent in English. Any differences between the original French version and the back-translated version were discussed by the translators and the developers. A back-translated English version was produced. Third, acceptability testing was performed on a small sample (from 6 to 10) of cancer caregivers. The understandability, misinterpretation, and acceptability were checked. Some terms were reworded and a new version was produced

- **Psychometric testing**

The latest version was validated in a larger sample of caregivers in order to test the psychometric properties and to check the reliability and sensitivity.

### Statistical analyses

The linguistic transcultural equivalence was ensured by the collaboration between the American physicians and the French developers. The psychometric testing was performed using the following procedure.

A confirmatory factor analysis (CFA) was performed using the LISREL model. The fit to the model was tested by computing the Root Mean Square Error of Approximation (RMSEA), and a value <0.08 was considered acceptable. Internal structural validity was assessed using item-dimension correlations: item internal consistency (IIC) was assessed by correlating each item with its scale (a correlation of 0.4 supported item internal consistency (IIC)), and item discriminant validity (IDV) was assessed by determining the extent to which items correlates with the dimension they are hypothesized to represent than with the other ones. Floor and ceiling effects were reported assessing the homogeneous repartition of the response distribution. For each dimension, internal consistency reliability was assessed using Cronbach’s alpha coefficient. A Cronbach’s alpha coefficient of at least 0.7 was expected for each scale [21]. The unidimensionality of each scale was assessed using Rasch analyses: item goodness-of-fit statistics (INFIT) and coefficient of Loevinger (H). INFIT statistics ranging between 0.7 and 1.2 and an H coefficient of at least 0.40 ensure that all the items of the scale tend to measure the same concept [22]. Differential item functioning (DIF) analyses were performed to compare the differences in item difficulties between the American caregivers and the French caregivers assessed in the CarGOQoL validation study [15].

To explore external validity, relations between the following dimensions were assessed using Pearson’s correlation coefficients (*r*): i) dimensions of CarGOQoL and the SF-36; and ii) dimensions of CarGOQoL and the VAS of burden/QoL. The underlying assumption was that the dimension scores of the CarGOQoL would better correlate with the scores of similar dimensions from the SF-36 than with dissimilar dimensions [15]. The discriminant validity was determined by assessing the associations between the CarGOQoL dimension scores and sociodemographic and clinical features. For qualitative variables, the mean dimension scores of the CarGOQoL were compared across patient groups that were expected to differ (e.g. gender, marital status, relationship, age classes) using Student’s *t* test. Quantitative variables (e.g. caregiving duration and disease duration), were analyzed using Pearson’s correlation coefficients. The underlying assumptions were derived from the initial validation of CarGOQoL [15]: women should report lower scores for emotional dimensions than men, caregivers who were single and who were children should report lower QoL, and patient’s disease duration and caregiving duration should be correlated to some dimensions of QoL.

Reproducibility was tested by assessing the test–re-test reliability using intraclass correlation coefficients (ICC) between the two successive assessments in stable caregivers who were defined in two ways: i) caregivers reporting no life events and ii) caregivers reporting no health changes of the patient. Sensitivity to change was tested through comparisons of the mean scores between the two successive assessments in caregivers who reported improved or worsened health statuses of the patient. Data analyses were performed using SPSS 11.0, MAP-R, and WINSTEP software.

## Results

### Sample characteristics

The study sample included 87 American caregivers of cancer patients. Table 1 details the different characteristics of the caregivers and patients. The mean (standard deviation) age of caregivers was 60 (standard deviation 11) years. In 65 % of cases, the caregiver was the spouse of the patient. The median caregiving duration was 8 years (interquartile 3–30). The most frequent locations of the patients’ cancer were breast and lung.

**Table 1:** Baseline characteristics

		*N* = 87
1. Caregivers		
Gender	Women	58 (68.2)
	Men	27 (31.8)
Age (years)	Mean ± SD^a^	60.0 ± 11.2
Living	With a partner	72 (84.7)
	Alone	13 (15.3)
Relationship with the patient	Partner	55 (64.7)
	Children	12 (14.1)
	Others^c^	18 (21.2)
Time of caregiving (months)	Median [IQR]^b^	8 [3–30]
2. Patients		
Gender	Women	43 (51.2)
	Men	41 (48.8)
Age (years)	Mean ± SD^a^	65.0 ± 12.8
Cancer location	Breast	23 (26.4)
	Lung	22 (25.3)
	Head and neck	10 (11.5)
	Hematologic	8 (9.2)
	Others^d^	24 (27.6)
Stage	0–1	12 (14.6)
	2	10 (12.2
	3	17 (20.7)
	4	43 (52.4)
Metastasis		45 (54.9)
Disease duration (months)	Median [IQR]^b^	11 [6–44]
WHO PS	0	50 (59.5)
	> = 1	34 (40.5)

^a^ SD standard deviation

^b^ IQR interquartile range

^c^ friend (5), brother/sister (4), parent (4), unknown (5)

^d^ urogenital (12), digestive (7), thyroid (1), knee (1), unknown primary (3)

### Construct validity and internal structural validity

The structure was confirmed using CFA, which showed a reasonable fit (RMSEA at 0.800). There were no differences in the DIF results between the American individuals and the French sample [15] for all of the items. All of the correlations of each item with its contributive dimension and with the other dimensions are presented in Table 2, which shows overlapping of some dimensions. Floor and ceiling effects were considered satisfactory (the ceiling effects of 3 dimensions were higher than 25 % of the Burden, Administration and finances, and Social support dimensions). Six dimensions of the CarGOQoL showed satisfactory internal consistency (Cronbach’s alpha: 0.71–0.87). Eight dimensions showed a satisfactory scalability. The Burden and the Relationship with healthcare dimensions (Burden, item 8 “… been embarrassed to be the only person to provide assistance”; Relationship with healthcare, item 10 “… been reassured by the health care providers” and item 11 “… felt that your role as caregiver was recognized by health care providers”) showed an INFIT statistics outside the acceptable ranges. All of the results are provided in Table 2.

**Table 2:** Dimensions’ characteristics of CargoQoL

Dimension (Items)	N	Mean ± SD^a^	IIC min - max	IDV min - max	% floor effect	% ceiling effect	Alpha	INFIT min-max
Psychological well being (4)	85	47,7 ± 22,0	0.67–0.78	−0.16–0.64	0	2.6	0.87	0.78–1.28
Burden (4)	85	83,8 ± 18,7	0.23–0.82	−0.13–0.55	0	29.5	0.79	0.73–2.06
Relationship with healthcare (3)	85	74,1 ± 17,0	0.19–0.42	−0.18–0.38	0	10.3	0.57	0.62–1.38
Administration and finances (3)	83	80,8 ± 20,5	0.42–0.60	−0.11–0.48	0	30.8	0.71	0.77–1.23
Coping (3)	84	68,3 ± 20,2	0.37–0.51	−0.11–0.55	0	11.5	0.64	0.84–1.22
Physical well being (4)	84	69,4 ± 21,0	0.56–0.68	−0.21–0.67	0	6.4	0.81	0.79–1.24
Self-esteem (2)	85	74,1 ± 20,7	0.62–0.62	−0.15–0.22	0	20.5	0.75	0.97–1.13
Leisure time (2)	85	54,8 ± 20,3	0.45–0.45	−0.14–0.48	0	1.3	0.60	0.89–0.99
Social support (2)	85	72,7 ± 22,0	0.61–0.61	0.04–0.33	0	25.6	0.76	0.95–1.02
Private life (2)	80	50,6 ± 22,8	0.15–0.15	−0.17–0.47	1.3	5.1	0.26	0.97–1.04
Index	78	67,6 ± 11,8						

M ± SD mean ± standard deviation; ICC item internal consistency; IDV item discriminant validity; Alpha Cronbach’s alpha; INFIT Rasch statistics

^a^ scores ranging from 0 to 100; the higher the score, the better the QoL

### External validity and discriminant validity

The concepts covered by the CarGOQoL and the SF-36 do not systematically overlap. In particular, some specific dimensions of CarGOQoL, including Relationship with healthcare and Self-esteem, were not correlated with the SF-36 dimensions. As expected, the ‘emotional-like’ dimensions of the CarGOQoL (Psychological well-being, Burden, and Coping) were moderately to highly correlated with the ‘emotional-like’ dimensions of SF-36 (Role emotional, Mental health, and the Mental composite score). Leisure and Private life were moderately correlated with the Social functioning score of SF-36. The Burden score was significantly correlated with the visual analogic scale of Burden. The index was significantly correlated with the VAS of QoL. All correlations are detailed in Table 3.

**Table 3:** Correlations between CargoQoL dimensions and other self-reported measures

		PsWB	B	RHC	AF	COP	PhWB	SE	LEI	SS	PL	Index
SF-36	Physical functioning	0,216^*^	0,139	0,103	0,164	0,177	0,207	0,028	0,241^*^	0,269^*^	0,266^*^	0,284^*^
	Social functioning	0,668^**^	0,623^**^	0,154	0,454^**^	0,514^**^	0,578^**^	0,017	0,514^**^	0,259^*^	0,469^**^	0,702^**^
	Role physical	0,372^**^	0,308^**^	0,040	0,309^**^	0,409^**^	0,446^**^	−0,040	0,334^**^	0,320^**^	0,344^**^	0,480^**^
	Role emotional	0,618^**^	0,438^**^	0,129	0,290^**^	0,528^**^	0,635^**^	0,160	0,371^**^	0,313^**^	0,394^**^	0,642^**^
	Mental health	0,722^**^	0,436^**^	0,098	0,339^**^	0,600^**^	0,587^**^	0,268^*^	0,478^**^	0,264^*^	0,338^**^	0,671^**^
	Vitality	0,535^**^	0,386^**^	0,090	0,252^*^	0,436^**^	0,583^**^	0,166	0,526^**^	0,373^**^	0,277^*^	0,580^**^
	Bodily pain	0,304^**^	0,181	−0,013	0,345^**^	0,168	0,427^**^	0,005	0,447^**^	0,357^**^	0,280^*^	0,411^**^
	General health	0,366^**^	0,293^**^	−0,005	0,340^**^	0,389^**^	0,511^**^	0,055	0,430^**^	0,252^*^	0,294^**^	0,440^**^
	PCS SF36	0,124	0,081	−0,001	0,189	0,111	0,217^*^	−0,097	0,254^*^	0,245^*^	0,214	0,198
	MCS SF36	0,721^**^	0,545^**^	0,140	0,338^**^	0,567^**^	0,635^**^	0,240^*^	0,488^**^	0,239^*^	0,374^**^	0,703^**^
QoL	VAS	0,545^**^	0,518^**^	−0,023	0,348^**^	0,551^**^	0,636^**^	0,088	0,620^**^	0,376^**^	0,304^**^	0,669^**^
Burden	VAS	−0,232^*^	−0,622^**^	0,013	−0,317^**^	−0,295^**^	−0,249^*^	0,040	−0,289^**^	−0,086	−0,260^*^	−0,396^**^

*PsWB* psychological well being, *B* burden, *RHC* relationship with healthcare, *AF* administration and finances, *COP* coping, *PhWB* physical well being, *SE* self-esteem, *LEI* leisure time, *SS* social support, *PL* private life; range [0–100]; higher score indicated higher QoL

SF-36 dimensions, PCS SF36 physical composite score, MCS SF36 mental composite score; range [0–100]; higher score indicated higher QoL

VAS QoL, range [0–10], higher score indicated higher QoL; VAS Burden, range [0–10]; higher score indicated higher burden

*p < 0.05, **p < 0.01, ***p < 0.001

The discriminant validity of CarGOQoL was assessed using the clinical and sociodemographic characteristics (Table 4). Women reported significantly lower scores in the Psychological well-being dimension. Caregivers living with a partner reported significantly better QoL in 2 dimensions: Administration and finances and Coping. Caregivers who were children and who were younger reported significantly lower scores in 3 and 2 dimensions. The age and gender of the caregivers were negatively linked to the caregivers’ QoL in some dimensions.

**Table 4:** Comparisons (mean ± standard deviation) and correlations (r) of CarGOQoL scores with respect to caregivers’ and patients’ characteristics

	PsWB	B	RHC	AF	COP	PhWB	SE	LEI	SS	PL	Index
Caregiver’s gender											
Women	42,67 ± 21,40	82,50 ± 19,94	76,50 ± 17,67	81,39 ± 20,34	67,10 ± 21,61	66,44 ± 20,68	75,21 ± 20,87	53,01 ± 20,18	71,55 ± 21,93	50 ± 24,28	66,26 ± 12,07
Men	58,71 ± 19,54	86,65 ± 15,67	69,13 ± 14,58	79,62 ± 21,35	70,98 ± 17,19	75,69 ± 20,82	71,75 ± 20,68	58,79 ± 20,45	75,46 ± 22,59	51,92 ± 19,90	70,52 ± 10,90
*p* value	**0,001**	0,441	**0,035**	0,582	0,432	**0,040**	0,386	0,149	0,455	0,615	0,123
Caregiver living with a partner											
No	42,78 ± 20,38	72,75 ± 24,52	78,52 ± 12,48	66,02 ± 26,23	56,41 ± 18,36	63,46 ± 19,90	77,88 ± 19,86	49,03 ± 24,18	61,53 ± 26,74	47,5 ± 26,87	60,85 ± 11,74
Yes	48,66 ± 22,33	85,82 ± 16,90	73,37 ± 17,67	83,57 ± 18,27	70,53 ± 19,96	70,51 ± 21,21	73,43 ± 20,97	55,90 ± 19,56	74,82 ± 20,71	51,07 ± 22,39	68,68 ± 11,55
*p* value	0,297	0,077	0,334	0,018	**0,017**	0,183	0,452	0,226	0,074	0,599	0,084
Relationship status											
Spouse	51,78 ± 21,78	87,84 ± 13,73	72,72 ± 16,23	83,79 ± 17,46	71,75 ± 17,91	71,87 ± 21,33	74,31 ± 18,54	58,63 ± 17,98	74,09 ± 21,89	49,76 ± 22,12	69,41 ± 11,18
Child	34,89 ± 22,36	67,01 ± 27,27	77,43 ± 20,75	68,93 ± 21,75	62,5 ± 25	63,02 ± 22,36	76,04 ± 24,69	45,83 ± 26,29	71,87 ± 22,69	43,18 ± 21,91	59,14 ± 13,00
Other	44,09 ± 19,58	82,75 ± 19,86	76,38 ± 17,20	79,16 ± 26,23	62,03 ± 22,36	66,31 ± 19,07	72,22 ± 25,20	49,30 ± 20,77	69,44 ± 23,17	58,59 ± 24,88	67,36 ± 11,35
*p* value	0,023	**0,024**	0,428	0,109	0,172	0,252	0,839	**0,049**	0,718	0,277	0,052
Caregiver’s age class											
40 – 59 years	41,81 ± 19,55	81,49 ± 20,33	75,48 ± 16,16	79,16 ± 19,06	66,86 ± 20,53	67,87 ± 18,22	75 ± 19,66	49,12 ± 17,54	75,87 ± 20,30	50 ± 24,04	66,36 ± 10,48
>59 years	53,86 ± 22,97	86,21 ± 16,77	72,81 ± 17,94	82,52 ± 22,11	69,91 ± 20,14	71,03 ± 23,78	73,21 ± 22,01	60,71 ± 21,48	69,64 ± 23,61	51,28 ± 21,80	69,06 ± 13,04
*p* value	**0,017**	0,173	0,444	0,210	0,516	0,239	0,769	**0,013**	0,238	0,773	0,322
Patient’s gender											
Women	48,20 ± 22,22	82,99 ± 21,35	71,80 ± 16,31	80,75 ± 20,69	66,27 ± 19,41	70,93 ± 21,85	71,51 ± 22,38	54,94 ± 22,35	72,67 ± 23,50	50 ± 21,18	67,41 ± 11,85
Men	46,49 ± 21,69	84,90 ± 15,87	76,82 ± 17,72	81,66 ± 20,34	70,41 ± 21,42	67,34 ± 20,33	76,52 ± 18,99	54,87 ± 18,51	73,47 ± 20,76	51,28 ± 24,96	67,96 ± 12,05
*p* value	0,729	0,695	0,198	0,805	0,398	0,353	0,356	0,920	0,956	0,992	0,972
Patient’s age class											
18 – 70 years	49,38 ± 21,05	86,14 ± 16,85	75,77 ± 15,38	83,95 ± 19,21	70,37 ± 18,57	71,06 ± 18,30	74,30 ± 20,23	56,48 ± 20,56	75,23 ± 20,81	50 ± 25,37	69,09 ± 11,15
>70 years	43,75 ± 23,15	79,93 ± 21,57	71,52 ± 19,81	75,89 ± 21,91	64,36 ± 23,24	65,73 ± 25,47	73,33 ± 22,19	52,08 ± 20,25	69,16 ± 24,06	51,78 ± 18,23	64,91 ± 12,96
*p* value	0,219	0,159	0,405	0,096	0,160	0,511	0,916	0,319	0,282	0,681	0,117
Patient’s disease duration											
r	0,061	−0,110	−0,168	0,007	−0,030	0,024	−0,070	−0,094	−0,299**	−0,264*	−0,187
	0,582	0,317	0,127	0,952	0,785	0,832	0,524	0,396	**0,006**	**0,019**	0,104
Caregiving duration											
r	−0,010	−0,091	−0,109	−0,137	−0,018	−0,076	0,016	−0,302**	−0,413**	−0,204	−0,248*
*p* value	0,929	0,423	0,336	0,233	0,878	0,505	0,885	**0,006**	**0,000**	0,079	**0,034**

*PsWB* psychological well being, *B* burden, *RHC* relationship with healthcare, *AF* administration and finances, *COP* coping, *PhWB* physical well being, *SE* self-esteem, *LEI* leisure time, *SS* social support, *PL* private life, range [0–100]; higher score indicated higher QoL

Bold values: *p*-value<0.05

### Reproducibility and sensitivity to change

The numbers of patients defined as stable based on the absence of self-reported positive/negative events in their life between 2 assessments were 17 on day- 30 and 19 in day- 90; the numbers of patients defined as stable based on the absence of self-reported health changes between 2 assessments were 39 on day- 30 and 29 on day- 90. The test-retest reliability at day- 30 was satisfactory showing an ICC ranging from 0–53 to 0.94, except for Relationship with healthcare and Private life (0.46 and 0.48, respectively). The reliability on day- 90 was also satisfactory, with an ICC higher than 0.50 for all dimensions except Relationship with healthcare and Self-esteem. All of these results are presented in Table 5.

**Table 5:** Reproducibility at day- 30 and day- 90

	Day- 30		Day- 90	
	No life events^a^	No health changes^b^	No life events^a^	No health changes^b^
Dimension	*N* = 17	*N* = 39	*N* = 19	*N* = 29
Psychological well being	0.64	0.57	0.65	0.54
Burden	0.88	0.74	0.80	0.60
Relationship with healthcare	0.53	0.46	0.43	0.60
Administration and finances	0.88	0.78	0.73	0.81
Coping	0.77	0.72	0.66	0.92
Physical well being	0.84	0.60	0.87	0.69
Self-esteem	0.67	0.74	0.48	0.59
Leisure time	0.88	0.72	0.70	0.69
Social support	0.63	0.52	0.53	0.58
Private life	0.75	0.48	0.60	0.49
Index	0.94	0.65	0.80	0.82

*ICC* Intraclass correlation coefficients

^a^ stable caregivers defined as no positive or negative changes in caregivers’ lives

^b^ stable caregivers defined as a patient’s stable health status

Sensitivity to change was tested on a sub-sample in which the patients’ health statuses either improved or worsened. The questionnaire was not sensitive to the change in the patient’s health status, except in the Leisure time dimension at day- 30 and in the Psychological well-being, Relationship with healthcare, Social support, and Administration and finances dimensions at day- 90. All these details are presented in Table 6.

**Table 6:** Sensitivity to change at day- 30 and day- 90: patient’s health status evolution reported by the caregiver

	Day- 30		Day- 90	
	Improved	Worsened	Improved	Worsened
	*N* = 20	*N* = 19	*N* = 24	*N* = 18
Dimension	Delta^a^	Delta^a^	Delta^a^	Delta^a^
Psychological well being	−6.67	4.28	−11.46**	9.19*
Burden	0.63	8.88	1.91	5.02
Relationship with healthcare	9.58	4.63	6.60*	4.17
Administration and finances	−2.91	3.43	−4.69	−7.03*
Coping	−3.33	3.24	−4.86	6.62
Physical well being	−0.31	4.51	0.78	7.99
Self-esteem	7.50	3.29	0	−0.78
Leisure time	8.13*	4.61	4.69	−0.74
Social support	5.00	1.97	9.89*	−8.82
Private life	0.66	10.29	4.67	3.57
Index	2.21	3.61	0.75	4.00

^a^ Delta: change between day-0 and day-i (score day-0 – score day-i)

*p < 0.05, **p < 0.001

### Acceptability

The median time for completion of the questionnaire was 6 min, with an interquartile range of 4 to 11. The missing data were very low (less than 0.5 %).

## Discussion

The CarGOQoL is a well-designed and well-validated questionnaire assessing QoL of cancer caregivers. The CarGOQoL was developed in France, and we reported the validation of the American version of the CarGOQoL in the United-States. To answer the scientific and regulatory requirements that are specific to this field, the development and linguistic validation should be based on a unique methodology acknowledged by health authorities, ethics’ committees, and researchers in the field [20]. The rigorous linguistic validation process that we used in this work ensures that the original concepts and content validity were retained, and that cross-cultural differences related to health between different countries were taken into account. The absence of DIF supports the good quality of the translation.

The availability of an American version of CarGOQoL will enable clinical investigators to incorporate an assessment of the CarGOQoL into their studies. Providing multiple language versions of a questionnaire allows researchers to pool data from different countries in multinational studies, to compare scores between countries and to establish norms. From this American version, other versions should be provided (such as an English version for United Kingdom and Australia). In this case, the standard process consists of a slighter cultural adaptation process. The American version will be considered the “mother” language, which will be adapted to the cultural and linguistic context of the target country.

Compared to other instruments assessing QoL of caregivers of cancer patients, the CarGOQoL has at least two interesting specificities. First, the questionnaire is designed to reflect the exclusive point of view held by caregivers themselves obtained from face-to-face, semi-structured interviews based on guidelines from the literature [23]. The questionnaire allows for the identification of specific dimensions, such as Self-esteem, that focus on positive aspects of caregiving [2], or Private life, which is not addressed in other questionnaires. Other questionnaires generally were developed by combining mixed points of views from caregivers, patients and experts and did not assess these specific dimensions [13, 14]. The American linguistic validation process was performed using the original French version in close collaboration with the French developers. This rigorous approach ensured that the translation faithfully reflected the original concepts in the initial questionnaire. Second, the CarGOQoL was initially validated on a large heterogeneous group of cancer caregivers, which included partners, parents and children. This accurately represents cancer patient relationships and captures all aspects of caregiver QoL. A large majority of previous studies focused on specific family relationships, such as spouses, parents, or children. Finally, the low rate of missing data and the short time of completion assure future use of this measure and make the CarGOQoL fully compatible with clinical practice.

The psychometric properties of the American version of the CarGOQoL can be considered satisfactory. Three items had INFIT statistics outside the range [0.7-1.2]. This range is applicable in the case of the development of a new test, but is larger in the case of an existing test, ranging from 0.5 to 1.5 [24]. In accordance with this interpretation, item 8 (“… been embarrassed to be the only person to provide assistance”) was the only item with an unacceptable range. We hypothesized that “assistance” is not identically understood by an American population compared to a French population. In the case of an existing test, the overfitting needs no action [25]. The study design allowed us to assess core psychometric properties, such as reproducibility and sensitivity to change, which are two core psychometric properties of a measuring instrument [26, 27]. This study showed that the CarGOQoL may be incorporated into longitudinal studies to detect a meaningful QoL change in cancer caregivers. As a specific QoL instrument that focuses on particular life problems, the CarGOQoL is sensitive enough to detect and quantify small changes. In accordance with the US Food and Drug Administration and the European Medicines Agency which encourages the use of QoL assessments, the CarGOQoL may be more appropriate than generic questionnaires due to its better ability to discern QoL differences in cancer caregivers. This measure could be used to monitor response to any intervention. An examination of responsiveness requires longitudinal data collection and is, therefore, rarely used in studies reporting the validation of QoL questionnaires. Finally, environmental barriers have been described [28] to explain why QoL measures have not been routinely implemented in clinical practice and clinical research. A great asset of a QoL questionnaire is its acceptability. The low rate of missing data and the short time of completion ensure the future use of this measure and make the CarGOQoL fully compatible with clinical practice.

The demographics of the French and American samples varied slightly. The American sample has a similar sex-ratio to the French sample used in the validation study of the initial French version of CarGOQoL. The American caregivers were older than the French caregivers (60+/− 11 vs. 52 +/− 14 [15]) and reported a shorter duration of caregiving. The majority of American caregivers were partners. Children and parents were underrepresented compared to the French sample. Indeed, the French sample contained an important contingent of caretakers who were predominantly parents due to the large number of haematological cancers affecting young patients in the French sample.

Some limitations should be considered. The major limitation is the small sample size, which requires replication of these findings in larger groups of caregivers. Likewise, some indicators of internal structural validity were sometimes not optimal. The degree to which the American structure matches the initial French structure should also be quantified. This quantification has already be applied using suitability indices [29]. These indices, which were produced from decision rules to define satisfactory properties according to appropriate standards [23, 30], allow for a more objective determination of the suitability or unsuitability of different structures.

## Conclusion

Despite some non-optimal indicators of validity, the American version of the CarGOQoL constitutes a useful, clinical instrument to measure QoL in caregivers of cancer patients in the United States. Further linguistic validation will be done using the English version of CarGOQoL for caregivers in the United Kingdom, Canada, and Australia to provide appropriate translations that are culturally relevant and conceptually equivalent to the original.
